# Immunophenotyping invasive breast cancer: paving the road for molecular imaging

**DOI:** 10.1186/1471-2407-12-240

**Published:** 2012-06-13

**Authors:** Jeroen F Vermeulen, Aram SA van Brussel, Petra van der Groep, Folkert HM Morsink, Peter Bult, Elsken van der Wall, Paul J van Diest

**Affiliations:** 1 Department of Pathology, University Medical Center Utrecht, Utrecht, The Netherlands; 2 Division of Internal Medicine and Dermatology, University Medical Center Utrecht, Utrecht, The Netherlands; 3 Department of Pathology, Radboud University Nijmegen Medical Centre, Nijmegen, The Netherlands

**Keywords:** Invasive breast cancer, Tumor markers, Optical imaging, Immunohistochemistry, Antibody panel

## Abstract

**Background:**

Mammographic population screening in The Netherlands has increased the number of breast cancer patients with small and non-palpable breast tumors. Nevertheless, mammography is not ultimately sensitive and specific for distinct subtypes. Molecular imaging with targeted tracers might increase specificity and sensitivity of detection. Because development of new tracers is labor-intensive and costly, we searched for the smallest panel of tumor membrane markers that would allow detection of the wide spectrum of invasive breast cancers.

**Methods:**

Tissue microarrays containing 483 invasive breast cancers were stained by immunohistochemistry for a selected set of membrane proteins known to be expressed in breast cancer.

**Results:**

The combination of highly tumor-specific markers glucose transporter 1 (GLUT1), epidermal growth factor receptor (EGFR), insulin-like growth factor-1 receptor (IGF1-R), human epidermal growth factor receptor 2 (HER2), hepatocyte growth factor receptor (MET), and carbonic anhydrase 9 (CAIX) 'detected' 45.5% of tumors, especially basal/triple negative and HER2-driven ductal cancers. Addition of markers with a 2-fold tumor-to-normal ratio increased the detection rate to 98%. Including only markers with >3 fold tumor-to-normal ratio (CD44v6) resulted in an 80% detection rate. The detection rate of the panel containing both tumor-specific and less tumor-specific markers was not dependent on age, tumor grade, tumor size, or lymph node status.

**Conclusions:**

In search of the minimal panel of targeted probes needed for the highest possible detection rate, we showed that 80% of all breast cancers express at least one of a panel of membrane markers (CD44v6, GLUT1, EGFR, HER2, and IGF1-R) that may therefore be suitable for molecular imaging strategies. This study thereby serves as a starting point for further development of a set of antibody-based optical tracers with a high breast cancer detection rate.

## Background

In The Netherlands, the lifetime risk to develop breast cancer increased in the last decades from 1 in 10 in 1989 to 1 in 7 in 2003 [1]. In parallel, the annual number of newly diagnosed cases of breast cancer rose to over 13,000 in 2008 [2]. This makes breast cancer the most commonly diagnosed female cancer in The Netherlands. Despite this increase in incidence, the number of deaths due to breast cancer has remained stable in the last decades, with annually around 3,300 deaths in The Netherlands in the period 1989–2008 [3]. Early detection by mammographic population screening has likely contributed to this, leading to diagnosis of smaller, often non-palpable breast cancers and ductal carcinoma *in situ* (DCIS) lesions [4,5]. Nevertheless, mammography is not optimally sensitive and specific, especially in younger patients and patients with dense breasts [6-11]. Ultrasonography and magnetic resonance imaging (MRI) have been shown to contribute to early detection of breast cancer, as has positron emission tomography (PET) imaging, but these three imaging devices also have their limitations [12].

Molecular optical imaging with near-infrared fluorescent (NIRF) probes holds promise here [13]. First, the spectral properties (emission wavelengths between 700–900 nm) of the fluorescent tracers result in low background (auto)fluorescence [14]. Second, the detection can be highly sensitive and specific and third, it enables to detect tumors up to centimeters deep in tissue [15]. Fourth, no protective measures are required since no ionizing radiation is emitted [16], and fifth, NIRF probes can be conjugated to highly specific targeted molecules such as antibodies, antibody fragments, peptides, or protease activatable substrates to increase the specificity of the signal in the tumor as reviewed by Pleijhuis et al. [17].

Several molecular targets have been suggested to be suitable for optical detection of breast cancer such as the epidermal growth factor receptor (EGFR) [18], vascular endothelial growth factor (VEGF) [13,19], and (human epidermal growth factor receptor 2) HER2 [20,21]. In addition, hypoxia up-regulated surface antigens like glucose transporter 1 (GLUT1) and carbonic anhydrase 9 (CAIX) that are expressed in about half of invasive breast cancers [22] and also in DCIS [23] and therefore might be valuable targets. Since NIRF antibodies will not be easily internalized, intracellular molecular targets relevant for optical detection of breast cancer have so far been ignored.

However, no single molecular target is expressed in all invasive breast cancers and at the same time provides adequate signal-to-noise ratio to the normal breast. For screening purposes a panel of probes, i.e. antibodies or antibody fragments will likely be necessary. Because development of such antibody-based probes is labor-intensive and costly, we set out to screen for expression of a selected set of candidate targets on tissue microarrays containing 483 cases of human invasive breast cancer, in search of the minimum antibody panel that would be suitable for detection of most breast cancers *in vivo* by molecular imaging.

## Methods

### Patients

The study population was derived from the archives of the Departments of Pathology of the University Medical Center Utrecht, Utrecht, and the Radboud University Nijmegen Medical Centre, Nijmegen, The Netherlands. These comprised 483 cases of invasive breast cancer (operated between 1997 and 2007), of which 340 cases were part of a consecutive series (operated between 2003–2007). The series was enriched with a small consecutive series of lobular breast cancers and a consecutive series of 23 cases with a BRCA germline mutation as previously described [24].

Histological grade was assessed according to the Nottingham scheme [25], and mitotic activity index (MAI) was assessed as before [26]. From representative donor paraffin blocks of the primary tumors, tissue microarrays were constructed by transferring tissue cylinders of 0.6 mm (3 cylinders per tumor) from the tumor area, determined by a pathologist based on haematoxylin-eosin stained slides, using a tissue arrayer (Beecher Instruments, Sun Prairie, WI, USA) as described before [27]. Normal breast tissue was obtained from patients that underwent mammoplasty (and thus had no tumor at all). In case of matched tumor and normal tissue, we analyzed normal tissue in paraffin blocks that did not contain any tumor and thus were far away from the tumor. The use of anonymous or coded left over material for scientific purposes is part of the standard treatment contract with patients in The Netherlands [28]. Ethical approval was not required.

### Immunohistochemistry

Immunohistochemistry was carried out on 4 μm thick sections for a panel of potential molecular membrane bound targets known to be expressed in a frequency of >10% in breast cancer. These were partly highly tumor specific, meaning that they have no or low intensity staining of the normal breast tissue (GLUT1, EGFR, insulin-like growth factor-1 receptor (IGF-1R), HER2, CAIX, hepatocyte growth factor receptor (MET)). We also included less tumor-specific, meaning that are known to have moderate or high intensity staining of the normal breast tissue (Mucin 1 (MUC1), CD44v6, Mammaglobin, transferrin receptor (TfR), carbonic anhydrase 12 (CAXII)), since cancers have usually increased cellularity compared to the normal breast and could thereby also provide adequate signal-to-noise in tumors compared to the normal breast.

After deparaffination and rehydration, endogenous peroxidase activity was blocked for 15 min in a buffer solution pH5.8 containing 0.3% hydrogen peroxide. After antigen retrieval, i.e. boiling for 20 min in 10 mM citrate pH6.0 (for progesterone receptor (PR), CD44v6, GLUT1, CAIX, MET, TfR, and CAXII), Tris/EDTA pH9.0 (estrogen receptor α (ERα), HER2, IGF1-R, MUC1, and Mammaglobin) or Prot K (0.15 mg/ml) for 5 min at room temperature (EGFR), a cooling off period of 30 min preceded the primary antibody incubation. CD44v6 (clone VFF18, BMS125 Bender MedSystems, Austria) 1:500; ERα (clone ID5, DAKO, Glostrup Denmark) 1:200; PR (clone PgR636, DAKO) 1:100; HER2 (SP3, Neomarkers, Duiven, The Netherlands) 1:100; GLUT1 (A3536, DAKO) 1:200; CAIX (ab15086, Abcam, Cambridge, UK) 1:1,000; IGF1-R (NB110-87052, Novus Biologicals, Cambridge, UK) 1:400; TfR (13–6800, Invitrogen, Breda, The Netherlands) 1:300; MUC1 (EMA, M1613 clone E29, DAKO) 1:400; Mammaglobin (clone 304-1A5, DAKO) 1:100; CAXII (HPA008773, Sigma Aldrich, Zwijndrecht, The Netherlands) 1:200 were incubated for 1 h at room temperature. Primary antibodies against EGFR (clone 31 G7, Zymed, Invitrogen) 1:30; MET (18–2257, Zymed, Invitrogen) 1:100 were incubated overnight at 4°C. All primary antibodies were diluted in PBS containing 1% BSA.

The signal was amplified using Powervision poly-HRP anti-mouse, rabbit, rat (DPVO-HRP, Immunologic, Duiven, The Netherlands) or the Novolink kit (Leica, Rijswijk, The Netherlands) (in the case of EGFR) and developed with diaminobenzidine, followed by counterstaining with haematoxylin, dehydration in alcohol and mounting.

### Scoring of immunohistochemistry

All stainings were compared to normal breast tissue and scored as positive when a clear membranous staining was seen and when the expression in the tumor was clearly higher than in the normal breast tissue. All stainings were scored using the DAKO/HER2 scoring system for membranous staining. Scores 2+ and 3+ were considered as positive except for HER2 where only a score of 3+ was considered positive. Due to the strong intra-tumor heterogeneity of Mammaglobin expression, scoring was performed by estimating the percentage of positive tumor cells, considering cancers with more than 35% of the membrane stained tumor cells as positive. All scoring was done by a single experienced pathologist (PJvD) who was blinded to patient characteristics and results of other stainings. To take tumor-heterogeneity between the tumor cores into account, the average score per tumor was calculated and used for analyses. Only in case of GLUT1 and CAIX, the tumor was classified as positive when a single core showed positivity. In this study a maximum of 3 missing stainings per patient was allowed, these stainings were considered as negative in the analyses. This potentially results in underestimation of the percentage positivity of a marker.

Based on ERα, PR, and HER2 immunohistochemistry, tumors were classified as luminal (ERα and/or PR positive), HER2-driven (ERα-, PR-, HER2+), triple negative (ERα-, PR-, HER2-) or basal (ERα-, PR-, HER2-, EGFR+), the immunohistochemical surrogate [29] of the original Sorlie/Perou classification [30].

### Immunofluorescence for quantification of protein expression in tumor and normal breast tissue

Several of the evaluated molecular membrane targets (CD44v6, MUC1, TfR, Mammaglobin, and CAXII) are known to be expressed to some extent in the normal breast epithelium. In order for these targets to be useful for breast cancer screening by optical imaging, the signal to background ratio needs to be high enough to be discriminative. We therefore performed immunofluorescence with these antibodies to allow quantification of expression ratios between normal breast and cancer tissue of four randomly selected patients by image analysis.

Immunofluorescence was performed as described above for immunohistochemistry, except that the primary antibodies were detected by incubation with Goat-anti-mouse/rabbit Alexa555 (1:1,000, Invitrogen) for 1 h at room temperature, followed by 4,6-Diamidine-2-phenylindole dihydrochloride (DAPI) counterstaining and mounting with Immumount (Thermo Scientific, Etten-Leur, The Netherlands).

Representative images of normal breast and breast cancers from the same sections were taken using identical settings at 20x magnification using a Leica DMI4000b inverted bright-field/fluorescence microscope.

### Image analysis of tumor expression versus normal breast tissue

Conventional immunohistochemical slides were digitalized for image analysis using a digital slide scanner (Aperio Technologies Inc., Vista, CA, USA). Of each patient four representative areas of normal and tumor tissue were selected and the average membrane intensity was calculated with the IHC membrane algorithm (Aperio, v8.001). As the signal-to-noise ratio *in vivo* is determined by the difference in expression between cancer and normal cells as well as by cellularity, the number of cells in the selected area was obtained from the algorithm. Tumor-to-normal ratio was calculated as (membrane intensity*cellularity/area) of the tumor/(membrane intensity*cellularity/area) of normal tissue.

Tumor-to-normal ratios of the fluorescently labeled antibodies were calculated with ImageJ using the median intensity scores. Values are expressed as the average tumor/normal ratio ± SEM.

Based on experience in radiology with the blood-pool agent indocyanine green in studies assuming a leaky vessel model [31,32], and from studies using NIRF labeled trastuzumab/bevacizumab in mouse models [33], a tumor-to-normal ratio larger than 3 was considered to be sufficient for optical imaging.

### Statistics

Statistical analysis was performed using IBM SPSS Statistics version 18.0 (SPSS Inc., Chicago, IL, USA). Associations between categorical variables were examined using the Pearson’s Chi-square test. P-values <0.05 were considered to be statistically significant.

## Results

To investigate the most promising combination of markers suitable for imaging, we studied the expression of a panel of membrane markers in our study population that comprised 319 (66.0%) invasive ductal, 126 (26.1%) invasive lobular, and 38 (7.9%) invasive breast cancers with other histology. Other clinicopathological characteristics are shown in Table 1.

**Table 1:** Clinicopathological characteristics of 483 invasive breast cancer patients studied for expression of selected membrane markers

**Feature**	**Grouping**	**N or value**	**%**
Age (years)	Mean	60	
	Range	28 to 88	
Histological type	Invasive ductal cancer	319	66.0
	Invasive lobular cancer	126	26.1
	Others	38	7.9
Tumor size (cm)	≤2	206	42.7
	>2 and ≤5	219	45.3
	>5	49	10.1
	Not available	9	1.9
Histological grade	1	89	18.4
	2	169	35.0
	3	219	45.4
	Not available	6	1.2
Lymph node status	Negative ^*^	225	46.6
	Positive ^**^	232	48.0
	Not available	26	5.4

^*^: negative = N0 or N0(i+); ^**^: positive = ≥N1mi (according to TNM 7^th^ edition, 2010).

**Figure 1:**
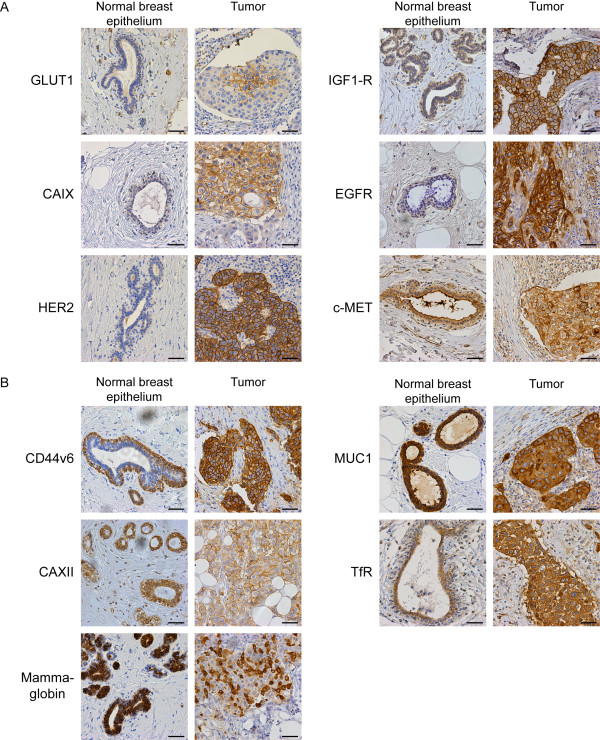
**Membrane marker expression in normal breast epithelium and breast cancer.** Images of representative breast cancer cases with the corresponding normal breast epithelium that were scored as positive. **A.** Expression of tumor-specific markers with low or no expression in normal breast epithelium. **B.** Expression of membrane markers that are also expressed in normal breast tissue. The intensity in the normal breast epithelium was classified as moderate or high. Size bar equals 50 μm.

Representative pictures of immunohistochemistry for the highly tumor-specific molecular membrane targets are shown in Figure 1a. The most widely expressed tumor-specific protein in our cohort was GLUT1, positive in 20.3% of the cancers, followed by EGFR (17.4%), IGF-1R (12.8%), HER2 (10.4%), CAIX (9.5%), and MET (8.9%). The less tumor-specific targets MUC1 (90.7%), CD44v6 (63.8%), Mammaglobin (16.8%), TfR (14.5%), and CAXII (8.7%) were in general more frequently expressed than the tumor-specific targets (Table 2). Representative pictures of immunohistochemistry for the less tumor-specific molecular membrane targets are shown in Figure 1b.

**Table 2:** Frequency of expression by immunohistochemistry of tumor-specific and less tumor-specific membrane markers in breast cancers

**Target**	**Positive**		**Negative**		**Missing**	
	**N**	**%**	**N**	**%**	**N**	**%**
HER2	50	10.4	432	89.4	1	0.2
EGFR	84	17.4	395	81.8	4	0.8
MET	43	8.9	423	87.6	17	3.5
IGF1-R	62	12.8	400	82.8	21	4.3
GLUT1	98	20.3	360	74.5	25	5.2
CAIX	46	9.5	414	85.3	25	5.2
TfR	70	14.5	402	83.2	11	2.3
CD44v6	308	63.8	160	33.1	15	3.1
CAXII	42	8.7	426	88.2	15	3.1
Mammaglobin	81	16.8	382	79.1	20	4.1
MUC1	438	90.7	26	5.4	19	3.9

### Detection rate of combinations of highly tumor-specific molecular targets in relation to grade, molecular and histological type

**Figure 2:**
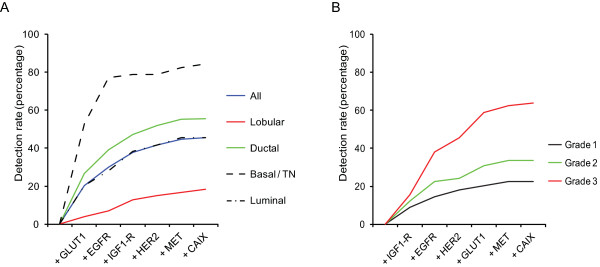
**Detection rate of tumor-specific membrane markers for detection of breast cancer.** Detection rate of highly tumor-specific membrane markers for detecting luminal, HER2-driven, basal/triple negative ductal breast cancers, and lobular breast cancers. The detection rate of tumor-specific markers for detection of breast cancer plotted as the positivity of the marker in combination with all preceding markers.

Because the frequency of expression (further denoted 'detection rate') of individual highly tumor-specific markers did not exceed 20.3% of the cases, we examined several combinations of markers by sequential addition of markers to the expression of GLUT1, the most widely expressed highly tumor-specific marker. GLUT1 in combination with EGFR resulted in 30.0% positive cases, GLUT1/IGF1-R in 28.8%, GLUT1/HER2 in 27.7%, GLUT1/MET in 25.2%, and GLUT1/CAIX in 22.3% positive cases. The panel GLUT1, EGFR, HER2, IGF1-R, MET, and CAIX resulted in 45.5% positive cases, although the contribution of CAIX and MET was minimal (Figure 2A).

Clear differences were found between histological subtypes of breast cancer (Table 3). Lobular carcinomas hardly expressed any of the tumor-specific membrane targets present in the panel compared to ductal carcinomas (detection rate 18.3% vs. 55.5%, p < 0.001). Within the group of lobular carcinomas, pleomorphic lobular carcinomas expressed more membrane targets than classical lobular carcinomas (detection rate 26.8% vs. 8.6%, p = 0.034). Within the group of ductal carcinomas, the basal/triple negative (TN) and HER2-driven ductal cancers expressed more frequently hypoxia markers or growth factor receptors than luminal-type ductal cancers (detection rate 84.2% vs. 45.0%, p < 0.001) (Table 4). Therefore the panel EGFR, MET, HER2, GLUT1, CAIX, and IGF1-R detected 84.2% of the basal/TN ductal breast cancers compared to 45.0% of the luminal-type, and 18.3% of the lobular breast cancer cases (Figure 2A, Tables 3 and 4). Because the markers included in our panel are associated with an aggressive phenotype and poor prognosis, we evaluated the detection rate of our panel in relation to grade (Figure 2B). Low grade (grade 1) tumors had a detection rate of 22.5% for this panel, in contrast to 33.7% of grade 2 and 63.9% of grade 3 tumors (p < 0.001). This indicates that the panel with tumor-specific antigens is less sensitive for detecting luminal-type, lobular, and low grade/well-differentiated tumors when applied for imaging strategies.

**Table 3:** Expression of a panel of membrane markers in various histological types of breast cancer

**Target**	**Ductal (319 cases)**		**Lobular (126 cases)**		**Other (38 cases)**	
	**N**	**%**	**N**	**%**	**N**	**%**
HER2	43	13.5	4	3.2	3	7.9
EGFR	71	22.3	4	3.2	9	23.7
MET	34	10.7	4	3.2	5	13.2
IGF1-R	48	15.0	7	5.6	7	18.4
GLUT1	85	26.6	5	4.0	8	21.1
CAIX	38	11.9	2	1.6	6	15.8
TfR	53	16.6	10	7.9	7	18.4
CD44v6	197	61.8	82	65.1	29	76.3
CAXII	30	9.4	12	9.5	1	2.6
Mammaglobin	44	13.8	34	27.0	3	7.9
MUC1	218	88.1	119	94.4	38	100

**Table 4:** Expression of membrane markers in molecular subtypes of ductal breast cancer

**Target**	**Luminal (242 cases)**		**HER2-driven (20 cases)**		**Basal/TN (57 cases)**	
	**N**	**%**	**N**	**%**	**N**	**%**
HER2	23	9.5	20	100	0	0.0
EGFR	25	10.3	11	55	35	61.4
MET	21	8.7	4	20	9	15.8
IGF1-R	41	16.9	2	10	5	8.8
GLUT1	49	20.2	6	30	30	52.6
CAIX	11	4.5	4	20	23	40.4
TfR	33	13.6	5	25	40	70.2
CD44v6	148	61.8	9	45	15	26.3
CAXII	28	11.6	1	5	1	1.8
Mammaglobin	39	16.1	4	20	1	1.8
MUC1	213	88.0	20	100	48	84.2

### Molecular targets that are expressed in normal breast tissue have sufficient signal-to-noise to detect lobular and luminal-type breast cancer

Since lobular and luminal-types of breast cancer appeared to hardly express tumor-specific antigens, antigens that are less tumor-specific are required for their detection. Like with tumor-specific markers, variation between histological and molecular subtypes was observed for TfR, Mammaglobin, and CAXII. Luminal-type ductal cancers and lobular cancers expressed significantly more CAXII (10.5% vs. 2.3%, p = 0.017) and Mammaglobin (19.9% vs. 5.9%, p = 0.002) compared to HER2-driven and basal/TN ductal cancers (Tables 3 and 4). TfR expression in lobular and luminal type ductal cancers was significantly lower than in HER2-driven and basal/TN cancers (11.9% vs. 27.9%, p < 0.001). For MUC1 and CD44v6, no differences in expression were found between lobular and ductal cancer (Tables 3 and 4).

Due to the expression of less tumor-specific antigens in the normal breast epithelium (Figure 1B), the signal-to-noise ratio (or tumor-to-normal) needs to be sufficiently discriminating to be applicable for imaging strategies. We determined therefore the tumor-to-normal ratio in a quantitative manner by image analysis of digital slides, considering a 3-fold tumor-to-normal ratio as sufficient. Image quantification using conventional IHC showed that the intensity of the staining was dependent on the cellularity of the tumor as expected. This resulted in tumor-to-normal ratios of 4.8 ± 0.56, 2.3 ± 0.27, 1.2 ± 0.095, 4.6 ± 0.62, and 2.4 ± 0.88 for CD44v6, MUC1, Mammaglobin, CAXII, and TfR, respectively.

**Figure 3:**
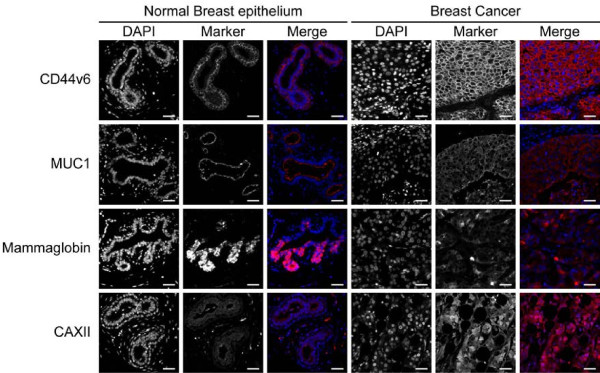
**Quantitation of expression levels of less tumor specific markers using immunofluorescence.** Expression levels of less tumor-specific membrane markers (CD44v6, MUC1, Mammaglobin, and CAXII) as determined by immunofluorescence resulted in staining patterns in normal breast epithelium and positive tumors comparable to conventional immunohistochemistry (Transferrin Receptor was not detectable). Size bar equals 25 μm.

Since conventional immunohistochemistry is not necessarily quantitative, we also performed immunofluorescence using directly fluorescently labeled antibodies. The results were comparable with conventional immunohistochemistry (Figure 3) resulting in tumor-to-normal ratios of 3.93 ± 0.14, 2.74 ± 0.46, 1.54 ± 0.11, and 1.66 ± 0.066 for CD44v6, MUC1, Mammaglobin, and CAXII, respectively. TfR expression was not detectable using immunofluorescence. Thereby, CD44v6 was the only less tumor-specific marker consistently meeting the required 3-fold tumor-to-normal ratio.

### Detection rate of combined highly and less tumor-specific molecular targets

Including TfR, Mammaglobin, and MUC1 to the panel of highly tumor-specific markers GLUT1, MET, EGFR, IGF1-R, CAIX, and HER2 increased the detection rate from 45.5% to 49.8% (TfR), 56.4% (Mammaglobin), and 98.1% (MUC1), respectively. However, of these markers, only CD44v6 reached a sufficiently high tumor-to-normal ratio (see above), so adding CD44v6 to the panel of highly specific markers therefore realistically increased the overall detection rate to 80.1%. When CD44v6 was included, removal of CAIX or MET from the panel had no influence on the detection rate.

**Figure 4:**
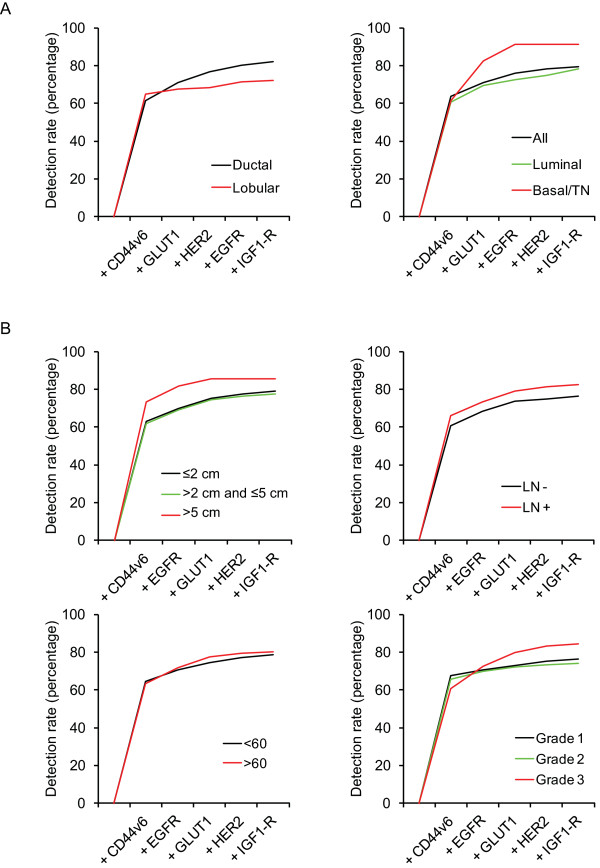
**Optimal combination of membrane markers for detection of breast cancer correlated with clincopathological characteristics.A.** The contribution of each tumor-specific and less tumor-specific membrane marker in the optimal panel for detection of breast cancer. **B.** The detection rate of the panel with respect to several clinicopathological features.

Especially the luminal-type ductal and lobular breast cancers were better detected by including CD44v6. Upon addition of CD44v6, the detection rate rose from 45.5% to 78.9% for luminal-type cancers, from 18.3% to 72.2% for the lobular breast cancers, and from 84.2% to 90.0% for basal/TN ductal breast cancers (Figure 4A). Moreover, the detection rate of the panel was not dependent on grade (76.4%, 74.0%, and 84.5% for grade 1, grade 2, and grade 3 tumors, respectively), tumor size (79.1%, 77.6%, and 85.7% for tumors ≤2 cm, >2 and ≤5 cm, and >5 cm, respectively), lymph node status (76.2% for lymph node negative, and 82.7% for lymph node positive cases), or age (78.8% for patients <60 years and 80.1% for patients >60 years) (Figure 4B).

Therefore, the optimal combination of membrane-expressed proteins to target by molecular imaging seemed to consist of CD44v6, GLUT1, EGFR, HER2, and IGF1-R by which about 80% of invasive breast cancers are predicted to be detectable.

## Discussion

The aim of this study was to identify the minimum panel of membrane markers that may be suitable for detection of invasive breast cancer by molecular imaging. In order to determine this combination, we stained TMAs consisting of 483 clinical specimens of invasive breast cancer by immunohistochemistry. Based on the expression profiles in the normal breast tissue, we defined highly tumor-specific (no or low staining of the normal breast tissue) and less tumor-specific (moderate or high staining of the normal breast tissue) membrane targets. We found that the expression of highly tumor-specific targets (HER2, EGFR, GLUT1, CAIX, IGF1-R, and MET) is quite dependent on the tumor histology and molecular subtype: ductal cancers and in particular the basal/TN and HER2-driven subtypes express more frequently highly tumor-specific membrane targets than lobular cancers.

Because the individual tumor-specific markers are clearly not sensitive enough, application of a tumor-specific panel of tracers is required to detect all types of breast cancer. A panel of tumor-specific markers (GLUT1, EGFR, HER2, IGF1-R, MET, and CAIX) was in the present study able to 'detect' 45.5% of all cancers and 55.6% of ductal cancers. For lobular cancers and low-grade tumors, the panel was not very suitable because with detection rates of 18.3% and 22.5%, respectively. Addition of less tumor-specific markers theoretically increased the detection rate to 98.1% using MUC1, but of the less tumor specific markers only CD44v6 met the desired 3-fold tumor-to-normal tissue ratio measured by image analysis. When adding CD44v6 to the panel, 80.1% of all cancers could be ‘detected’ with at least one marker in a panel consisting of HER2, GLUT1, EGFR, IGF1-R, and CD44v6. CAIX and MET had no additional effect on the sensitivity of the panel once CD44v6 had been included.

Our estimation of positivity of breast cancers for our panel may have been conservative since we have been very stringent in calling expression positive, explaining why our rates of expression for GLUT1, CAIX, EGFR, MET, TfR, CAXII, and Mammaglobin are on the lower side compared to the literature [22,34-43]. Tumors with 1+ membrane staining were consistently considered negative as we expect that this level of staining provides insufficient signal-to-noise, but only *in vivo* studies can confirm this. Moreover, quantification of expression levels based on image analysis of immunohistochemical stainings may be hampered by the non-linear amplification of the signal during immunohistochemistry. For that reason we applied immunofluorescence of directly labeled antibodies for more reliable quantitation of protein expression. Tumor-to-normal ratios above 3 where only obtained when tumors are scored as DAKO 2+ or 3+ membranous staining. This justifies the predefined thresholds for calling tumors positive. Furthermore, cytoplasmic staining was ignored as imaging antibodies will not be easily internalized and will have to bind to receptors on the outside of the cancer cells. Lastly, using TMAs may have resulted in slight underestimation of GLUT1 and CAIX expression, because the expression is usually limited to hypoxic areas within the tumor [44,45].

Adding further candidate tumor markers may enable to improve the results of our panel of membrane related markers. For instance, biomarkers that are specifically expressed in the stroma of breast cancers like growth factors (e.g. VEGF) may be valuable.

This study provides information on the expression levels of membrane bound targets for imaging using paraffin embedded material of invasive breast cancers. To be suitable for breast cancer detection or screening, multiple steps have to be taken before tracer development and testing in (pre)clinical trials results in treatment of patients. However, the present study elucidates which targets might be most suitable based on the expression in cancer vs. normal breast tissue. One of the current challenges is specific detection of lobular breast cancers and DCIS, because these lesions are difficult to detect by mammography. DCIS was beyond the scope of the current paper, but for detection of lobular breast cancer CD44v6 is potentially quite useful.

Next to expression of target proteins, tumor perfusion and penetration of the tracer into the tumor could influence the signal for imaging. Further, affinity after labeling and half-life of the tracer in the human body determine the tumor-to-background ratio and thus the applicability of a tracer in a clinical setting. Based on preclinical studies using NIRF labeled trastuzumab and bevacizumab, the maximal tumor-to-background ratio was obtained 6 days post injection [33]. Optimizing this by reducing the half-life of the tracer would be beneficial for clinical practice.

The present study underlines that no single membrane marker probe is likely to detect all breast cancers by molecular imaging, and that a panel of least five probes may be required. So far, experience is however limited to maximally two different tracers at once. Barrett et al. [46] showed that two antibodies allowed to identify differences in tumor expression of HER2 and EGFR *in vivo*. When aiming to be just discriminative between tumor and normal, a panel of markers can be injected with the same probe attached to simplify imaging. Feasibility and toxicity of injecting a panel of markers require further *in vivo* experiments in mouse models.

## Conclusions

We studied which tumor membrane markers are most discriminating between invasive breast cancer and normal breast tissue in order to identify the minimal number of targeted probes needed for the highest possible breast cancer detection rate. We showed that 80% of all breast cancers express at least one of a panel of markers (CD44v6, GLUT1, EGFR, HER2, and IGF1-R) that therefore may be suitable for molecular imaging strategies. The present study thereby serves as a starting point for further development of a set of antibody-based optical tracers with high potential for detecting breast cancer.

## Competing interest

The authors declare that they have no competing interest.

## Authors’ contributions

JFV, ASAvB, PvdG, and FHMM performed the experiments. PB and PJvD provided the study material, analyzed and interpreted tumor pathology. PvdG, PB, EvdW, and PJvD critically reviewed the experiments and paper. JFV, ASAvB, and PJvD wrote the manuscript. All authors critically reviewed the report and approved the final version of the report for submission. The corresponding author (PJvD) had access to the primary data, took responsibility for accuracy and completeness of data reporting, and had final responsibility for the decision to submit for publication. All authors read and approved the final manuscript.

## Pre-publication history

The pre-publication history for this paper can be accessed here:

http://www.biomedcentral.com/1471-2407/12/240/prepub
